# The Orphan Working School, Haverstock Hill

**Published:** 1893-05-13

**Authors:** 


### THE ORPHAN WORKING SCHOOL, HAVERSTOCK HILL.

THE annual dinner in aid of the funds of this institution was held on Friday last in the Whitehall Rooms, Hotel Métropole. The chair was taken by Lord Battersea, and amongst those present were Louisa, Lady Ashburton, Lord Kinnaird, General Sir Redvers Buller, Sir Hugh Low, Sir Francis Osborne, Mr. B. Woodd-Smith (treasurer), and Mr. Algernon Coote (secretary). Lord Battersea, in proposing the toast of the evening, spoke of the good work that was being carried on by this institution. It had risen from small beginnings, and was now one of the largest, and ought to be one of the best-known, charities in London. Six hundred children were cared for at Haverstock Hill and Hornsey Rise, and none left the schools without fitting employment being found for them. £5,000 was now urgently needed, and he made a confident appeal for further support. Donations amounting to rather more than £2,000 were announced.

